# New thermodynamic activity-based approach allows predicting the feasibility of glycolysis

**DOI:** 10.1038/s41598-021-85594-8

**Published:** 2021-03-17

**Authors:** Thorsten Greinert, Kristina Vogel, Thomas Maskow, Christoph Held

**Affiliations:** 1 Laboratory of Thermodynamics, Department of Biochemical and Chemical Engineering, TU Dortmund University, Emil-Figge-Strasse 70, 44227 Dortmund, Germany; 2 Department of Environmental Microbiology, UFZ - Helmholtz Centre for Environmental Research, Permoserstr. 15, 04318 Leipzig, Germany

**Keywords:** Biocatalysis, Enzymes, Biochemistry, Biosynthesis, Catalysis, Chemical engineering, Physical chemistry, Chemical synthesis

## Abstract

Thermodynamic feasibility analyses help evaluating the feasibility of metabolic pathways. This is an important information used to develop new biotechnological processes and to understand metabolic processes in cells. However, literature standard data are uncertain for most biochemical reactions yielding wrong statements concerning their feasibility. In this article we present activity-based equilibrium constants for all the ten glycolytic reactions, accompanied by the standard reaction data (standard Gibbs energy of reaction and standard enthalpy of reaction). We further developed a thermodynamic activity-based approach that allows to correctly determine the feasibility of glycolysis under different chosen conditions. The results show for the first time that the feasibility of glycolysis can be explained by thermodynamics only if (1) correct standard data are used and if (2) the conditions in the cell at non-equilibrium states are accounted for in the analyses. The results here will help to determine the feasibility of other metabolisms and to understand metabolic processes in cells in the future.

## Introduction

Biotechnological routes offer several advantages over classical chemical routes to produce chemicals, such as higher reaction selectivity and milder reaction conditions, yielding an overall more sustainable processes^1^. Furthermore, such routes access a world full of new syntheses protocols using reactions that require the use of an enzyme or multiple enzymes in a complete metabolic pathway. However, finding such new syntheses routes is generally expensive due to high experimental effort. So-called thermodynamic feasibility analyses (TFA) help to determine whether a metabolic pathway is generally feasible or not from thermodynamic point-of-view. The feasibility largely depends on the composition of the reaction medium, temperature, pressure, and pH. The reason behind this multifaceted dependency is the interaction between the enzyme and the surrounding reaction medium as well as the influence of the reaction medium on the interactions of the metabolites^2, 3^. Thus, including thermodynamic constraints to develop new processes is promising; such constraints help to evaluate that thermodynamically unfeasible routes are excluded. For this purpose, the TFA tools need to be accurate, reliable, and robust even at cellular conditions. As metabolic networks are highly complex, the level of accuracy might be decisive for the establishment of an impactful TFA. Several research groups studied thermodynamic concepts to analyze biochemical reactions and networks, such as the Heinemann group^4, 5^, Heijnen group^6^, Hatzimanikatis group^7^, many based on the work from Alberty^8^. The eQuilibrator is among the most famous online tools to calculate fluxes using thermodynamic constraints^9^.

In literature the feasibility of reactions is determined using thermodynamic constraint data derived from metabolic concentrations. In a first step of such an approach concentration of metabolites at equilibrium are determined to access the standard Gibbs energy of reaction ${\Delta }^{R} g^{0}$. Afterwards, ${\Delta }^{R} g^{0}$ is used to calculate the thermodynamic driving force of a reaction, i.e. Gibbs energy of reaction ${\Delta }^{R} {\text{g}}$; this determines whether the reaction is feasible or not at the conditions under investigation.

1 $${\Delta }^{R} g = {\Delta }^{R} g^{0} + RTln\left( {\text{Q}} \right)$$

In Eq. (1) the $Q$ term describes the actual ratio of metabolite activities in the cell, i.e. $Q$ is a non-equilibrium variable. In the literature, the $Q$ term is often assumed to be the ratio of the metabolite concentrations; that is, activity coefficients of the metabolites are ignored in most analyses. Only some researchers used the extended Debye–Hückel equation, to estimate the activity coefficient associated with each metabolite species, e.g.^10, 11^. Further, it has been shown that the extended Debye–Hückel equation did strongly underestimate the non-ideality of the reaction mixtures^12^. The drawback of neglecting activity coefficients or using oversimplified models is that strongly concentration-dependent behavior of metabolic reactions cannot be described. The reason behind this are that the activity coefficients of the metabolites are not equal to one, neither is their ratio, which is required in the $Q$ term. Furthermore, the presence of crowders or salts even more impacts the activity coefficients. Neglecting activity coefficients of the metabolites ultimately leads to misinterpretations of feasibility studies.

Besides the $Q$ term, also the standard term plays an important role in the evaluation using Eq. (1). In the recent years, new standard data (especially ${\Delta }^{R}{g}^{0}$) for glycolytic reactions have been accessed by equilibrium concentrations^12–26^ combined with thermodynamic modeling^12, 17, 18, 22–24, 26^. In these works, it was proven that the equilibrium concentrations of glycolytic reactions strongly depend on the medium conditions. Other works from literature indeed agree with these observations. Cornell et al. performed such measurements for the phosphoglycerate kinase (PGK) reaction at 311.15 K and pH 7.0 and varied the Mg^2+^ concentration^27^. They found that the reaction equilibrium was shifted far to the right upon increasing the concentration of Mg^2+^ from 0.3 to 8.1 mM. Wangler et al. found that even the concentration of the metabolites strongly influenced the reaction equilibrium of the PGK reaction^24^, which was verified by measuring the equilibrium upon increasing substrate concentrations from 1 to 20 mM. That is, ${\Delta }^{R}{g}^{0}$ values cannot be derived simply by the experimentally observed metabolite concentrations at reaction equilibrium; the equilibrium position strongly depends on the medium conditions. Instead, activity coefficients are required to convert concentrations into activities, i.e. converting apparent equilibrium constants (often denoted ${K}_{c}$ or ${K}_{m}$) into the thermodynamic equilibrium constant ${K}_{a}$ that depends only on temperature and pressure. This is possible by using the activity-coefficient ratio of the metabolites ${K}_{\gamma }$ (${K}_{a}={K}_{m}\cdot {K}_{\gamma }$). Activity coefficients $\gamma$ are important as they describe molecular interactions between a metabolite with its surrounding medium. Exact values for $\gamma$ and ${K}_{\gamma }$ allow quantifying the influence of the reaction medium on the reaction equilibrium. A suitable tool to access $\gamma$ and ${K}_{\gamma }$ is the electrolyte Perturbed-Chain Statistical Associating Fluid Theory (ePC-SAFT)^28^. Wangler et al. determined ${K}_{\gamma }$ with ePC-SAFT at measured metabolite equilibrium concentration in order to calculate ${K}_{a}$ and an activity-based ${\Delta }^{R}{g}^{0}$ for the PGK reaction.

Certainly, most of the researchers in biochemistry are aware of the fact that activity coefficients are usually ignored; however, this assumption causes inconsistent use of thermodynamic equilibrium constants. Still, most of the TFA approaches are concentration-based; probably, the reason behind this is the fact that a tool to convert the classical concentration-based evaluation of Eq. (1) into the consistent thermodynamic activity-based treatment is not yet readily developed. Indeed, Maskow and von Stockar found that neglecting activity coefficients is the main reason for a failure of the description of glycolysis by TFA, followed by less reliable standard data^29^. The reason behind non-reliable standard data is again the assumption that activity coefficients do not play a role in the data reduction steps from the raw equilibrium data (typically concentrations) to the standard data (activities).

Thus, it is a key goal of this work to provide such a tool for all the ten glycolytic reactions, represented schematically in Figure S1. In this work, evaluating Eq. (1) based on thermodynamic activities instead of concentrations is discussed based on a tool that will be used to close that research gap. The so-obtained activity-based TFA approach using new standard data was applied in this study to demonstrate the feasibility of glycolysis using thermodynamically consistent data—the ultimate goal of the authors upon starting their research activities in that field in 2013.

## Results

### Standard Gibbs energy of reaction

In this section, the activity-based standard Gibbs energy of reaction values ${\Delta }^{R}{g}^{0}$ for all glycolytic reactions are provided, which are required for the calculation of ${\Delta }^{R}g$ and therefore the prediction of the feasibility of those reactions (see Eq. (1)). The standard enthalpy of reaction ${\Delta }^{R}{h}^{0}$ and ${\Delta }^{R}{g}^{0}$ of hexokinase reaction, glucose 6-phosphate (G6P) isomerase reaction, triosephosphate isomerase (TPI) reaction, glyceraldehyde 3-phosphate dehydrogenase (GAPDH) reaction, PGK reaction, enolase reaction and pyruvate kinase (PK) reaction and ${\Delta }^{R}{h}^{0}$ of phosphofructokinase (PFK) reaction, aldolase reaction and phosphoglycerate mutase (PGAM) reaction were used without further modifications from the respective sources. They are listed in Table 1. For the other reactions, standard data was determined in this work, which is presented in the following. In general, the tools required to determine standard data are (1) experimental equilibrium concentrations of the metabolites and (2) their activity coefficients at the respective equilibrium concentrations. The ratio of the equilibrium concentrations of the metabolites is further denoted ${K}_{c}$ or ${K}_{m}$ (molarity-based or molality-based), the activity-coefficient ratio is ${K}_{\gamma }$.

**Table 1:** Standard enthalpy of reaction of glycolysis $\Delta^{R} h^{0}$ and standard Gibbs energy of reaction of glycolysis $\Delta^{R} g^{0}$ at pH 7 with the respective sources.

Reaction	$\Delta^{R} h^{0}$		$\Delta^{R} g^{0}$(298.15 K)	
	kJ mol^−1^	Source	kJ mol^−1^	Source
Hexokinase	− 23.8 ± 0.7	^16^	− 17.8 ± 0.5	^17^
G6P isomerase	12.1 ± 0.2	^18^	2.94 ± 0.05	^18^
PFK	− 9.5	^19^ + ^20^^a^	− 9.3 ± 0.3	^13^ + ^19^ + this work^b^
Aldolase	48.97	^21^	22 ± 2	^14^ + ^21^ + this work^c^
TPI	18 ± 7	^22^	7.1 ± 0.3	^22^
GAPDH	4.6 ± 0.1	^23^	51.5 ± 0.4	^23^
PGK	− 49 ± 9	^24^	− 17.8 ± 0.2	^24^
PGAM	2 ± 5	^12^	− 5.8	^15^ + this work^d^
Enolase	27 ± 10	^12^	− 2.8 ± 0.2	^12^
PK	− 9.3	^25^	− 28 ± 1	^26^

^a^Value originally from Redman–Furey^19^; according to Goldberg et al.^20^ calorimetrically determined enthalpies (using different buffers at pH 9.0 and 298.15 K) were used to calculate ${\Delta }^{{\text{R}}} h^{0}$ = 9.5 kJ mol^−1^ with the change in the binding of ${\text{H}}^{ + }$ and enthalpies of ionization of the buffers.

^b–d^See text for more detail.

The ${\Delta }^{R}{g}^{0}$ of the PFK reaction was determined in this work based on the equilibrium-concentration ratio ${K}_{c}$= 2290 ± 270 from Hansen et al.^13^. It was determined at pH 8, 303.15 K, 33 mM Tris + HCl, 6.94 mM MgC${\mathrm{l}}_{2}$, 50 mM KCl, 1 mM dithiothreitol and 6 mM ${\left({\mathrm{NH}}_{4}\right)}_{2}$S${\mathrm{O}}_{4}$. Although the value from Hansen et al. is an equilibrium value, it is far away from standard state, which is the ideally diluted aqueous solution. This reference state requires that salts or other stabilizers must not be present in the solution, and that the metabolite concentrations approach zero. Certainly, this reference state can usually not be realized in experiments using enzymes. Thus, to access the standard state, ${\Delta }^{R}{g}^{0}$ and ${K}_{a}$, ePC-SAFT was used to predict activity coefficients, which describe the deviation from ideally diluted solution at the respective conditions. Further, it was assumed that concentration (mmol L^−1^) equals molality (mmol kg^−1^), and thus ${K}_{c}$ equals ${K}_{m}$ (i.e. $\rho$ of the solution is 1 kg L^−1^). As a result, ${K}_{\gamma }$ = 0.19 was obtained with parameters from Tables 2 and 3 considering all substances present in the reaction solution, except the enzyme and dithiothreitol. This yields ${K}_{a}$(pH 8, 303.15 K) = ${K}_{m}\cdot {K}_{\gamma }$, which was then used to calculate ${K}_{a}$(pH 7, 303.15 K) using the species distributions of fructose 6-phosphate (F6P), adenosine triphosphate (ATP), fructose 1,6-bisphosphate (FBP) and adenosine diphosphate (ADP) (see Table S1 for $p{K}_{\mathrm{A}}$ values). ${K}_{a}$(pH 7, 298.15 K) was calculated using ${\Delta }^{R}{h}^{0}$ =  − 9.5 kJ mol^−1^ from Table 1 and Eq. (5). ${K}_{a}$(pH 7, 298.15 K) finally yields ${\Delta }^{R}{g}_{\mathrm{PFK}}^{0}$ = − 9.3 ± 0.3 kJ mol^−1^ with Eq. (3).

**Table 2:** ePC-SAFT pure-component parameters applied in this work with the sources for the respective sets of parameters.

	$m_{i}^{{{\text{seg}}}}$	$\sigma_{i}$	${{u_{i} } \mathord{\left/ {\vphantom {{u_{i} } {k_{B} }}} \right. \kern-\nulldelimiterspace} {k_{B} }}$	$N_{i}^{{{\text{assoc}}}}$	${{\varepsilon^{{A_{i} B_{i} }} } \mathord{\left/ {\vphantom {{\varepsilon^{{A_{i} B_{i} }} } v}} \right. \kern-\nulldelimiterspace} v}$	$\kappa^{{A_{i} B_{i} }}$	$z_{i}$	Source
	–	Å	K	–	K	–	–	
Glucose	6.6260	2.9860	244.53	5 + 5	5000.0	0.1	–	^30^
ATP	50.1628	2.1398	164.92	7 + 7	862.4	0.0001	–	^17^
ADP	18.8255	2.3283	169.55	6 + 6	1285.5	0.0001	–	^17^
G6P	22.3290	2.2266	243.31	5 + 5	5000.0	0.1	–	^18^
F6P	35.5936	1.8100	198.49	5 + 5	5000.0	0.1	–	^18^
FBP	19.8735	2.2922	215.77	5 + 5	5000.0	0.1	− 3	^31^
DHAP	1.3472	4.1611	289.43	2 + 2	3614.4	0.1	− 1	^22^
GAP or 2-PG or 3-PG	3.1100	4.6600	322.02	5 + 5	501.2	0.0001	− 2	^24^
NA${\text{D}}^{ + }$	25.0875^a^	2.2714^a^	299.04	8 + 8	3557.3	0.001	–	^32^
NADH	27.3947	2.7559	380.52	8 + 8	3711.9	0.001	− 2	^23^
BPG	2.9053	2.3452	216.84	5 + 5	501.2	0.0001	− 4	^23^
PEP	12.0070	2.2000	407.27	2 + 2	5000.0	0.1	− 2	^12^
Pyruvate	18.7471	2.0812	141.02	2 + 2	0	0.04509	− 1	^33^
Water	1.2047	^b^	353.94	1 + 1	2425.7	0.04509	–	^34^
Tris	6.3730	2.7484	302.16	1 + 1	4786.9	0.02027	–	^18^
${\text{K}}^{ + }$	1	3.3417	200.00	–	–	–	+ 1	^35^
N${\text{a}}^{ + }$	1	2.8232	230.00	–	–	–	+ 1	^35^
${\text{H}}_{{3}} {\text{O}}^{ + }$	1	3.4654	500.00	–	–	–	+ 1	^35^
Tris-${\text{H}}^{ + }$	10.2047	2.4081	348.10	4 + 4	10,970.9	10^–6^	–	^18^
${\text{NH}}_{4}^{ + }$	1	3.5740	230.00	–	–	–	+ 1	^35^
${\text{Mg}}^{2 + }$	1	3.1327	1500.00	–	–	–	+ 2	^35^
C${\text{l}}^{{}}$	1	2.7560	170.00	–	–	–	− 1	^35^
${\text{H}}_{{2}}$P${\text{O}}_{{4}}^{ - }$	1	3.6505	95.00			–	− 1	^35^
HP${\text{O}}_{{4}}^{{2 - }}$	1	2.1621	146.02	–	–		− 2	^35^
${\text{SO}}_{4}^{2 - }$	1	2.6491	80.00	–	–	–	− 2	^35^

^a^Typo in the original reference from Wangler et al. The values given here must be used.

^b^$\sigma_{{{\text{water}}}}$ = 2.7927 + 10.11 *exp*(−0.01775 *T*) − 1.417 *exp*(−0.01146 *T*) from^34^.

**Table 3:** Binary interaction parameters $k_{ij}$ between metabolites and water, between ions and water or among ions used in this work.

Components	Binary interaction parameter		Source
$i$/$j$	$k_{ij}^{{{\text{slope}}}}$	$k_{ij}^{{T = 0 {\text{K}}}}$	
${\text{H}}_{3} {\text{O}}^{ + }$/water	0	0.25	^35^
N${\text{a}}^{ + }$/water	− 0.007981	2.37999	^35^
${\text{K}}^{ + }$/water	− 0.004012	1.3959	^35^
${\text{NH}}_{4}^{ + }$/water	0	0.064	^35^
C${\text{l}}^{ - }$/water	0	− 0.25	^35^
${\text{H}}_{{2}}$P${\text{O}}_{{4}}^{ - }$/water	0	0.25	^35^
${\text{Mg}}^{2 + }$/water	0	− 0.25	^35^
HP${\text{O}}_{{4}}^{2 - }$/water	0	0.25	^35^
S${\text{O}}_{{4}}^{2 - }$/water	0	0.25	^35^
${\text{K}}^{ + }$/C${\text{l}}^{ - }$	0	0.064	^35^
${\text{K}}^{ + }$/${\text{H}}_{{2}}$P${\text{O}}_{{4}}^{ - }$	0	0.018	^35^
${\text{K}}^{ + }$/HP${\text{O}}_{{4}}^{2 - }$	0	1.000	^35^
${\text{K}}^{ + }$/S${\text{O}}_{{4}}^{2 - }$	0	1.000	^35^
N${\text{a}}^{ + }$/C${\text{l}}^{ - }$	0	0.317	^35^
N${\text{a}}^{ + }$/${\text{H}}_{{2}}$P${\text{O}}_{{4}}^{ - }$	0	− 0.071	^35^
N${\text{a}}^{ + }$/HP${\text{O}}_{{4}}^{2 - }$	0	− 1.000	^35^
N${\text{a}}^{ + }$/S${\text{O}}_{{4}}^{2 - }$	0	− 1.000	^35^
N${\text{H}}_{4}^{ + }$/C${\text{l}}^{ - }$	0	− 0.566	^35^
N${\text{H}}_{4}^{ + }$/${\text{H}}_{{2}}$P${\text{O}}_{{4}}^{ - }$	0	− 1.000	^35^
N${\text{H}}_{4}^{ + }$/HP${\text{O}}_{{4}}^{2 - }$	0	− 0.556	^35^
N${\text{H}}_{4}^{ + }$/S${\text{O}}_{{4}}^{2 - }$	0	− 1.000	^35^
${\text{Mg}}^{2 + }$/C${\text{l}}^{ - }$	0	0.817	^35^
${\text{Mg}}^{2 + }$/S${\text{O}}_{{4}}^{2 - }$	0	− 1.000	^35^
${\text{H}}_{3} {\text{O}}^{ + }$/C${\text{l}}^{ - }$	0	0.654	^35^
Glucose/water	0.00024	− 0.1192	^30^
ATP/water	0	− 0.1719	^17^
ADP/water	0	− 0.1368	^17^
G6P/water	0	− 0.065	^18^
F6P/water	0	− 0.065	^18^
FBP/water	0	− 0.1011	^31^
DHAP/water	0	0	^22^
GAP/water	0.0020333	− 0.7064	^24^
NA${\text{D}}^{ + }$/water	0	− 0.074	^32^
NADH/water	0	− 0.056	^23^
BPG/water	0	0	^23^
PEP/water	− 0.005083	1.3316	^12^
Pyruvate/water	0	0.1601	^33^
Tris/water	0	− 0.047	^18^
Tris-${\text{H}}^{ + }$/water	0	− 0.061	^12^

${\Delta }^{R}{g}^{0}$ of the aldolase reaction was determined in this work based on equilibrium concentrations measured at pH 7, 311.15 K, 10 mM sodium phosphate buffer and 230 mM KCl by Veech et al.^14^. To obtain ${K}_{a}$, it was again assumed that concentration ${c}_{i}$ equals molality ${m}_{i}$ and ePC-SAFT was used to calculate ${K}_{\gamma }$ = 5.8, yielding ${K}_{a}$(pH 7, 311.15 K) with parameters from Tables 2 and 3 considering all substances present in the reaction solution, except the enzyme. Veech et al. used KCl to adjust ionic strength to 0.25 M. Thus, 230 mM KCl were considered for the ePC-SAFT prediction. Van 't Hoff equation (Eq. (5)) was then used to convert ${K}_{a}$(pH 7, 311.15 K) to ${K}_{a}$(pH 7, 298.15 K). The value ${\Delta }^{R}{h}^{0}$ = 48.97 kJ mol^−1^ from Table 1 was used for this step, which finally yielded ${\Delta }^{R}{g}_{\mathrm{Aldolase}}^{0}$ = 22 ± 2 kJ mol^−1^ with Eq. (3).

${\Delta }^{R}{g}^{0}$ of the PGAM reaction was determined in this work based on the equilibrium-molality ratio of metabolites at 303.15 K and pH 7 taken from Clarke et al.^15^. Therefore, ${K}_{m}$ was converted to 298.15 K with ${\Delta }^{R}{h}^{0}$ = 2 kJ mol^−1^ from Table 1 (please note that this value has an uncertainty of ± 5 kJ mol^−1^). It was assumed that activity coefficients of 3-PG and 2-PG are equal because of the isomeric character of the substances. Therefore, ${K}_{\gamma }$ was assumed to be equal to one, which yielded ${\Delta }^{R}{g}_{\mathrm{PGAM}}^{0}\hspace{0.17em}=\hspace{0.17em}$− 5.8 kJ mol^−1^.

**Figure 1:**
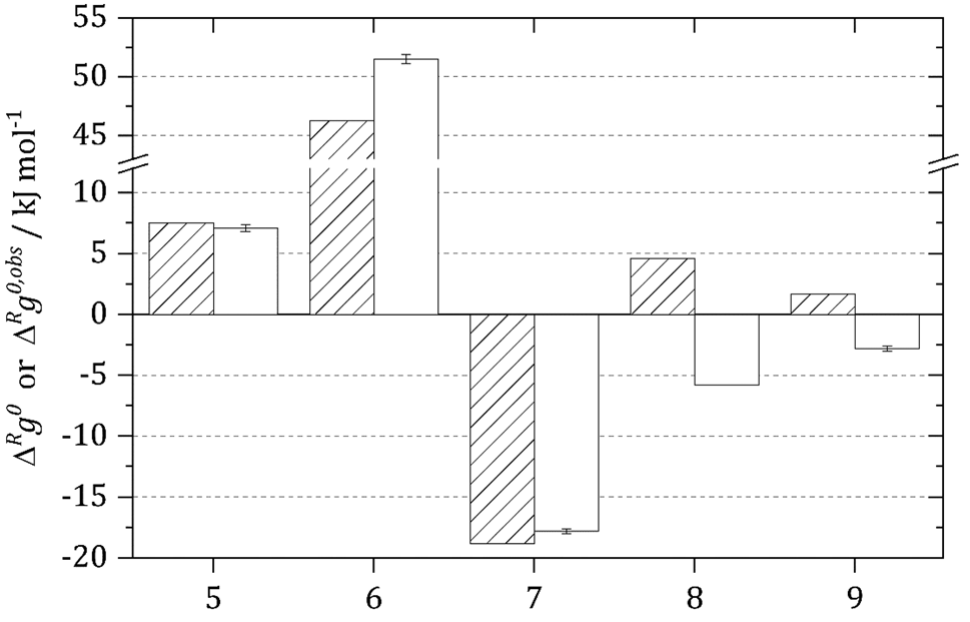
Comparison between the concentration-based standard Gibbs energy of reaction ${\Delta }^{R} g^{0,obs}$ (striped bars)^29^ and the activity-based standard Gibbs energy of reaction ${\Delta }^{R} g^{0}$ (white bars) of the glycolytic TPI reaction (5), GAPDH reaction (6), PGK reaction (7), PGAM reaction (8) and enolase reaction (9) at 298.15 K and pH 7.0 based on^12, 15, 22–24^. Please note that H^+^ was not considered for GAPDH reaction in the original source, but was added by us as this is a reaction participant.

Activity-based ${\Delta }^{R}{g}^{0}$ values listed in Table 1 might significantly differ (Fig. 1) from their concentration-based pendants ${\Delta }^{R}{g}^{0,obs}$ available in literature; this is important to note as the latter are typically used in TFA. ${\Delta }^{R}{g}^{0}$ and ${\Delta }^{R}{g}^{0,obs}$ might even show changes in the sign, which occurs e.g. for the PGAM reaction and for the enolase reaction. These differences directly influence the prediction of the feasibility of these reactions, as only ${\Delta }^{R}{g}^{0}$ must be used in Eq. (2). The use of ${\Delta }^{R}{g}^{0,obs}$ yields inconsistent results.

### Prediction of metabolic networks with TFA: thermodynamic activity-based approach versus classical concentration-based approach

**Figure 2:**
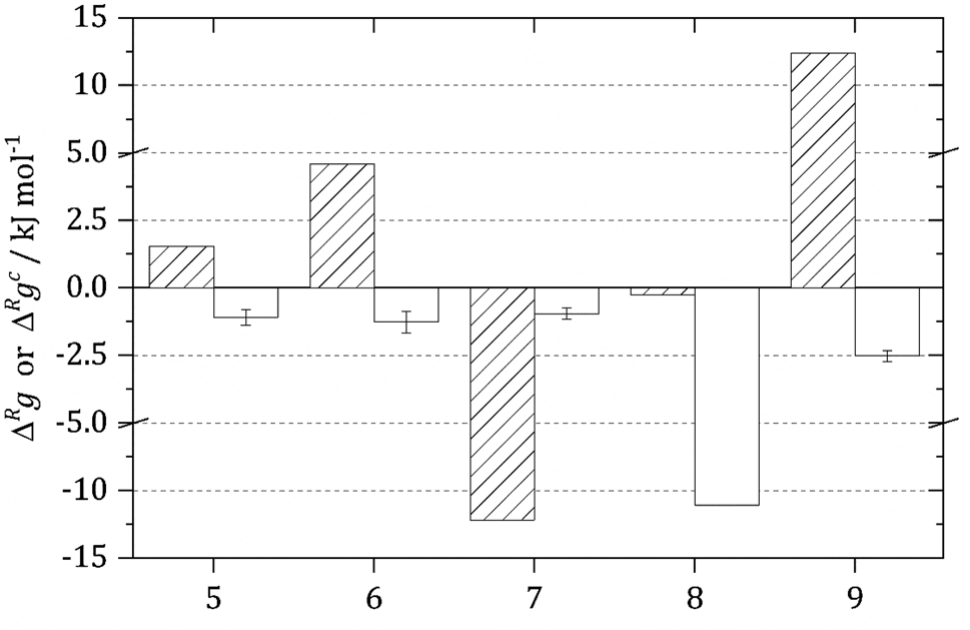
Comparison of the concentration-based Gibbs energy of reaction ${\Delta }^{R} g^{c}$ (striped bars) and the activity-based Gibbs energy of reaction ${\Delta }^{R} g$ (white bars) of the glycolytic TPI reaction (reaction 5), GAPDH reaction (reaction 6), PGK reaction (reaction 7), PGAM reaction (reaction 8) and enolase reaction (reaction 9) at 298.15 K, pH 7.0; reaction conditions listed in Table S2.

The activity-based approach accounts for molecular interactions that play a major role for the feasibility studies especially in complex intracellular media. A TFA was performed for glycolysis applying the activity-based approach, and it was compared to the classical concentration-based approach. Both, the classical TFA and the new activity-based TFA predict that reaction 1–4 and 10 occur spontaneously, i.e. ${\Delta }^{R}g$ for these reactions are negative. Hence, these reactions are not critical from the thermodynamic point-of-view. In the following, the results are discussed for reactions 5–9. The evaluation steps proposed in this work require that solving ${\Delta }^{R}g$ (see Eq. (2)) is performed applying activity-based values for ${\Delta }^{R}{g}^{0}$ (Table 1) and ${Q}_{a}$. Using ${\Delta }^{R}{g}^{0}$ or ${\Delta }^{R}{g}^{0,obs}$ (Fig. 1) in Eq. (1), the activity-based ${\Delta }^{R} g$ and its concentration-based pendant (the result of the classical approach, ${\Delta }^{R} g^{c}$) were determined, respectively. For both evaluations, exactly same reaction conditions (metabolite concentrations, T, pH, presence of salts) were considered; see Table S2 for reaction conditions. The comparison of both TFA analyses is shown in Fig. 2, yielding the new activity-based ${\Delta }^{R} g$ and the classical concentration-based ${\Delta }^{R} g^{c}$ for reaction 5–9. The values differ significantly resulting in partly even opposite results of the feasibility studies. Only the activity-based approach yields negative ${\Delta }^{R} g$ values for all the ten glycolytic reactions; only the activity-based TFA predicts that glycolysis is feasible under the given conditions. In contrast, the concentration-dependent TFA predicts that glycolysis will not take place as most of the reactions have positive ${\Delta }^{R} g$ values; however, this latter result contradicts experimental findings. This shows that only the activity-based approach allows performing meaningful TFA calculations.

In sum, the key to correct TFA prediction results of glycolysis is a thermodynamic activity-based evaluation. Thus, the activity-based procedure should be used in future to predict the feasibility of metabolic reactions. This approach further allows determining influences of the reaction medium on the glycolytic reactions based on molecular interactions between the medium and the substrate and product metabolites. These influences will be discussed in the following, using the activity-based approach only.

### Prediction of metabolic networks: TFA for glycolysis without versus with accounting for the medium influence on metabolite activity coefficients

**Figure 3:**
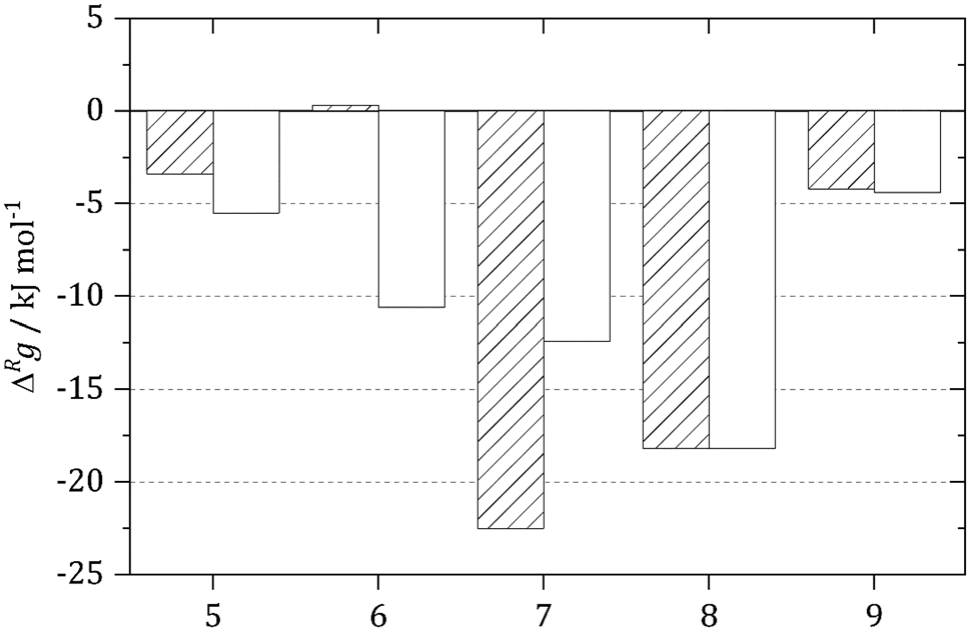
Comparison of the Gibbs energy of reaction ${\Delta }^{R} g$ determined considering substrates and products of the respective reaction for prediction of $Q_{\gamma }$ (striped bars) and ${\Delta }^{R} g$ determined considering all metabolites involved in glycolytic pathway (best-case concentrations for reaction participants + maximum concentration for the other metabolites within glycolysis, see Table S4) and 1 mmol kg^−1^ MgC${\text{l}}_{2}$ and 100 mmol kg^−1^ KCl for prediction of $Q_{\gamma }$ (white bars) of the glycolytic TPI reaction (5), GAPDH reaction (6), PGK reaction (7), PGAM reaction (8) and enolase reaction (9) at 298.15 K and pH 7.0.

*Step 1: Activity-based TFA without accounting for the medium influence on metabolite activity coefficients* In a first step, reaction conditions for each single reaction were applied only considering the presence of the substrate and product metabolites of the respective reaction, but neglecting the influence of salts and all metabolites of the other glycolytic reactions on the activity coefficients of the metabolites (reaction conditions **A** in Table S3). Further, a bunch of possible substrate concentrations are available from different literature sources. In this work, consistently maximum substrate and minimum product concentrations of the metabolites belonging to the respective reactions were considered. These were used to calculate $Q_{m}$ and to predict $Q_{\gamma }$; in Table S4 these minimum and maximum metabolite concentrations are listed. Using these conditions is helpful to determine reactions that will not occur under any reaction condition determined as plausible before as this choice represents an optimal best-case situation; still, it does not represent a cell where the reactions occur in the same reaction solution (neglecting compartmentalization here). The resulting $\Delta^{R} g$ values at 298.15 K are shown in Fig. 3 (striped bars). The results show that even at the chosen reaction conditions, the GAPDH reaction turned out to be unfeasible if only the substrates and products of the GAPDH reaction are considered for the prediction of $Q_{\gamma }$. The fact that $\Delta^{R} g^{0}$ ($\Delta^{R} h^{0}$) of the GAPDH reaction have estimated errors of 0.4 kJ mol^−1^ (0.1 kJ mol^−1^) is only little comfort because the reaction remains non-feasible at any reaction condition applied in this step even considering the uncertainty of these data behind. As optimal best-case conditions were applied for this study, positive $\Delta^{R} g$ means that the reaction is unfeasible also at any other substrate/product concentration ratio. For the other glycolytic reactions, negative $\Delta^{R} g$ values at 298.15 K and 310.15 K were found at reaction conditions **A**. That is, there are substrate/product concentration ratios within the minimum and maximum concentrations in Table S4, at which TFA predicted that these reactions occur spontaneously.

*Step 2: Activity-based TFA with accounting for the medium influence on metabolite activity coefficients* In step 2, the presence of all metabolites involved in the glycolysis pathway is considered for the prediction of $Q_{\gamma }$. Therefore, the maximum concentration of the metabolites not involved in the respective reaction and the substrate/product concentration ratios equal to step 1 were applied (see Table S4 for minimum and maximum metabolite concentrations). Since in a cell, where glycolysis occurs, there are also other substances like salts, additionally 1 mmol kg^−1^ MgC${\text{l}}_{2}$ and 100 mmol kg^−1^ KCl were considered in step 2 for the prediction of $Q_{\gamma }$—still at the substrate/product concentration ratios equal to step 1 and with accounting for the presence of all metabolites involved in the glycolytic pathway.

The resulting $\Delta^{R} g$ values at 298.15 K are shown in Fig. 3 (white bars). The driving force $\Delta^{R} g$ of most of the reactions are significantly greater for step 2 (Fig. 3) compared to step 1 (Fig. 2). The comparison of $\Delta^{R} g$ calculated applying reaction conditions **A** and **B** shows that especially the metabolites not involved in the respective reaction have strong effects on $\Delta^{R} g$. Metabolites and salts barely influence the enolase reaction, while they increases $\Delta^{R} g$ of the PGK reaction by 7 kJ mol^−1^ (but is still feasible) and decreases $\Delta^{R} g$ of the TPI reaction by 3 kJ mol^−1^. This shows that the strength of the influence of these metabolites and salts on $\Delta^{R} g$ depends on the chemical nature of the respective substrate and product metabolites and the interactions induced by specific forces such as charges or hydrogen bonding—all this is accounted for by the thermodynamic model ePC-SAFT.

A highly interesting result was found for the GAPDH reaction. $\Delta^{R} g$ of this reaction was positive, even at best-case conditions, applying reaction conditions **A** that neglect presence of other metabolites and salts on the activity-coefficient term of the metabolites $Q_{\gamma }$. Accounting for metabolites and salts (reaction conditions **B)** caused a shift of $\Delta^{R} g$ for the GAPDH reaction from positive to $\Delta^{R} g$ =  − 10.6 kJ mol^−1^ at 298.15 K and to $\Delta^{R} g$ =  − 11.4 kJ mol^−1^ at 310.15 K. That is, GAPDH becomes feasible under these more cytosolic-like conditions, while it is unfeasible at reaction conditions **A** (only reaction participants of the GAPDH reaction present in water). This shows that it is crucial to consider not only the metabolic reaction participants but also the metabolites of the other glycolytic reactions; i.e. in general all substances present in the reaction solution must be considered.

## Conclusions

In this study, a thermodynamic activity-based approach is presented that allows to correctly determine the feasibility of glycolysis under different conditions. Therefore, activity-based equilibrium constants for all the ten glycolytic reactions are required, which yield standard reaction data (standard Gibbs energy of reaction and standard enthalpy of reaction) that is now available for all the ten glycolytic reactions. New activity-based TFA calculations were performed with these data and the results show for the first time that it is possible to verify the feasibility of glycolysis. It was shown that this is only possible if (1) correct standard data are used and if (2) the conditions in the cell at non-equilibrium states are accounted for in the analyses. These new insights will allow to significantly reduce the experimental effort for the development of completely new biotechnological routes by means of TFA to a minimum extent. The classical concentration-based TFA causes misinterpretations about the feasibility of the reactions or pathways and cannot be used for this purpose, especially under cytosolic conditions, i.e. at high concentrations of metabolites or salts.

The activity-based approach additionally allows to better understand processes within cells by investigating influences of single reaction conditions like specific salts or concentrations of specific metabolites on single reactions and thus the complete metabolism. Such investigations might reveal and explain regulatory effects within cells and therewith yield a better understanding of cellular processes in general.

Further, an activity-based approach allows predicting the medium influence on the reaction progress; this can be adapted to biotechnological processes as well. The benefits of the activity-based approach to predict ${\Delta }^{R} g$ are obvious for the determination of the feasibility of single reactions and reaction pathways. However, the output of such an approach is a binary statement whether a certain reaction is thermodynamically feasible or not. It would also be interesting to know how fast a reaction is if it is feasible. That is, kinetic effects are important for the performance of a process in a cell or a bioreactor, and activity-based expressions for Michaelis constants are already available^36^. Thus, a combination of TFA applying the approach discussed here with kinetic studies of the reactions in a next step will be necessary in future works. We thus recommend applying the proposed activity-based approach for the design of new biotechnological processes in the future.

## Methods

### Reaction thermodynamics

Reactions with negative Gibbs energy of reaction $\Delta^{R} g$ occur spontaneously in the stated direction, while those with positive $\Delta^{R} g$ do not. $\Delta^{R} g$ is accessible by the standard Gibbs energy of reaction $\Delta^{R} g^{0}$ and the activity ratio of metabolites in the respective reaction solution $Q_{a}$:

2 $${\Delta }^{R} g = {\Delta }^{R} g^{0} + RTln\left( {Q_{a} } \right)\quad {\text{with}}\quad Q_{a} = \mathop \prod \limits_{i} a_{i}^{{v_{i} }}$$

$\Delta^{R} g^{0}$ is calculated from the thermodynamic equilibrium constant $K_{a}$:

3 $${\Delta }^{R} g^{0} = - RTln\left( {K_{a} } \right)$$

At known $Q_{a}$ and $K_{a}$ the pendants of these properties at non-standard conditions are accessible by activity coefficients:

4 $$Q_{\gamma } = \frac{{Q_{a} }}{{Q_{x} }}\quad {\text{and}}\quad K_{\gamma } = \frac{{K_{a} }}{{K_{x} }}$$

Knowing the activity coefficients allows to apply an activity-based approach based on reaction-medium-independent standard values ${\Delta }^{R} g^{0}$.

The temperature dependence of the thermodynamic equilibrium constant is described by the standard enthalpy of reaction ${\Delta }^{R} h^{0}$. In this work, according to the van ’t Hoff equation shown in Eq. (5), ${\Delta }^{R} h^{0}$ was calculated from the temperature dependence of $K_{a}$.

5 $$\left( {\frac{{dlnK_{a} }}{dT}} \right)_{{\text{p}}} = \frac{{{\Delta }^{R} h^{0} }}{{RT^{2} }}$$

Therefore, Eq. (5) was integrated assuming a temperature-independent ${\Delta }^{R} h^{0}$. This is a reasonable assumption for small temperature ranges as applied in this work (298.15–310.15 K).

### ePC-SAFT equation of state

The required activity coefficients for $Q_{\gamma }$ and $K_{\gamma }$ were predicted with ePC–SAFT^35, 37^. Within ePC-SAFT, the residual Helmholtz energy $A^{{{\text{res}}}}$ is calculated from different contributions:

6 $$A^{{{\text{res}}}} = A^{{{\text{hc}}}} + A^{{{\text{disp}}}} + A^{{{\text{assoc}}}} + A^{{{\text{ion}}}}$$

$A^{{{\text{hc}}}}$, $A^{{{\text{disp}}}}$, $A^{{{\text{assoc}}}}$, and $A^{{{\text{ion}}}}$ are contributions to the residual Helmholtz energy caused by hard-chain repulsion, dispersive interactions (van der Waals energy), associative interactions (e.g., hydrogen bonding forces), and Coulomb interactions (Debye–Hückel theory), respectively. To account for these contributions, five pure-component parameters are required for ePC-SAFT, see Table 2. The volume of the hard chains is described by the segment number $m_{i}^{{{\text{seg}}}}$ and the segment diameter $\sigma_{i}$. Dispersive interactions are described by the dispersion-energy parameter $u_{i} /k_{B}$ including the Boltzmann constant $k_{B}$. Associative interactions are described by the association-energy parameter $\varepsilon^{{A_{i} B_{i} }} /k_{B}$ and the association-volume parameter $\kappa^{{A_{i} B_{i} }}$. Additionally, the number of association sites $N_{i}^{{{\text{assoc}}}}$ must be chosen prior to modeling and the valence $z_{i}$ of an ion $i$ is required for $A^{{{\text{ion}}}}$. For mixtures, the binary interaction parameter $k_{ij}$ is used to correct for deviations of the combined dispersion-energy parameter from the geometric mean, see Table 3. Please note, that none of these parameters were fitted to reaction data. Rather, thermodynamic data of binary mixtures (metabolite + water) were used as input data to adjust the required ePC-SAFT parameters listed in Tables 2 and 3. The prediction method using ePC-SAFT then requires these parameters and one arbitrarily chosen equilibrium position at known conditions.

Derivation of $A^{{{\text{res}}}}$ with respect to density and mole fraction yields fugacity coefficients and activity coefficients of the substrates and products. The activity coefficients of the metabolites were calculated by the fugacity coefficient of the metabolite in the solution divided by the fugacity coefficient of the metabolite in an ideally diluted aqueous system:

7 $${\upgamma }_{{\text{i}}}^{*} = \frac{{\varphi_{i} }}{{\varphi_{i}^{inf} }}$$

## Supplementary Information

Supplementary Information
