# Supplementary material for: New thermodynamic activity-based approach allows predicting the feasibility of glycolysis
Source: Sci Rep. 2021 Mar 17;11:6125. doi: 10.1038/s41598-021-85594-8 (PMC7971085; doi:10.1038/s41598-021-85594-8)
Supplement: Supplementary file 1 — Supplementary Information [file 41598_2021_85594_MOESM1_ESM.pdf]

# A New Thermodynamic Activity-Based Approach Allows Predicting the Feasibility of Glycolysis.

*Thorsten Greinert[a], Kristina Vogel[b], Thomas Maskow[b], Christoph Held*[a]*

## Supplementary Information

[a] Laboratory of Thermodynamics, Department of Biochemical and Chemical Engineering, TU Dortmund, Emil-Figge-Strasse 70, 44227 Dortmund, Germany

[b] UFZ - Helmholtz Centre for Environmental Research, Dept. Environmental Microbiology, Leipzig, Permoserstr. 15, D-04318 Leipzig, Germany

* Correspondence: Christoph.Held@tu-dortmund.de (Christoph Held)

Table S1: $\boldsymbol{pK_A}$ and $\boldsymbol{pK_{Mg}}$ values for metabolites used in this work.

| Reaction | $pK_A$ or $pK_{Mg}$ |
|---|---|
| $H_2ATP^{2-} \rightleftharpoons HATP^{3-} + H^+$ | $pK_{A1}$ = 4.68 *(1)* |
| $HATP^{3-} \rightleftharpoons ATP^{4-} + H^+$ | $pK_{A2}$ = 7.60 *(1)* |
| $Mg_2ATP \rightleftharpoons ATP^{4-} + 2Mg^{2+}$ | $pK_{Mg1}$ = 2.69 *(1)* |
| $MgHATP^{-} \rightleftharpoons HATP^{3-} + Mg^{2+}$ | $pK_{Mg2}$ = 3.63 *(1)* |
| $MgATP^{2-} \rightleftharpoons ATP^{4-} + Mg^{2+}$ | $pK_{Mg3}$ = 6.18 *(1)* |
| $H_2ADP^{-} \rightleftharpoons HADP^{2-} + H^+$ | $pK_{A1}$ = 4.36 *(1)* |
| $HADP^{2-} \rightleftharpoons ADP^{3-} + H^+$ | $pK_{A2}$ = 7.18 *(1)* |
| $MgHADP \rightleftharpoons HADP^{2-} + Mg^{2+}$ | $pK_{Mg1}$ = 2.50 *(1)* |
| $MgADP^{-} \rightleftharpoons ADP^{3-} + Mg^{2+}$ | $pK_{Mg2}$ = 4.65 *(1)* |
| $HF6P^{-} \rightleftharpoons F6P^{2-} + H^+$ | $pK_{A1}$ = 6.27 *(1)* |
| $MgF6P \rightleftharpoons F6P^{2-} + Mg^{2+}$ | $pK_{Mg1}$ = 3.32 *(2)* |
| $H_2FBP^{2-} \rightleftharpoons HFBP^{3-} + H^+$ | $pK_{A1}$ = 6.05 *(1)* |
| $HFBP^{3-} \rightleftharpoons FBP^{4-} + H^+$ | $pK_{A2}$ = 6.65 *(1)* |
| $MgFBP^{2-} \rightleftharpoons FBP^{4-} + Mg^{2+}$ | $pK_{Mg1}$ = 3.75 *(2)* |

Table S2: Metabolite concentrations in µM (molarities assumed to be equal to molalities for any calculation). Values were determined by authors at different conditions in different organisms, see respective sources for more detailed information.

| metabolites | Minakami et al. 1966 *(11)* | Lehninger 1975 *(10)* | Maskow et al. *(7)*+ Jaklitsch et al. *(8)*+ Müller et al. *(9)* | Pissarra et al. 1997 *(4)*+ Führer et al. *(5)*+ Torres *(6)* | Stephanopoulos et al.1998 *(3)* |
|---|---|---|---|---|---|
| Glucose | 5000 | 5000 | | | |
| G6P | 38.5 | 83 | | | |
| F6P | 15.7 | 14 | | | |
| FBP | 7 | 31 | | | |
| DHAP | 17 | 138 | | | |
| GAP | 5.7 | 18.5 | | | |
| BPG | 0.4 | | | | |
| 3-PG | 68.5 | 118 | | | |
| 2-PG | 10 | 29.5 | | | |
| PEP | 17 | 23 | | | |
| Pyruvate | 85 | 51 | | | |
| ATP | 1830 | 1850 | 570-1740 | 500 | 7900 |
| ADP | 180 | 138 | 230-840 | 75 | 1040 |
| $P_i$ | 1000 | 1000 | | 500 | 7900 |
| $NAD^+$ | | | 650-1200 | 1310 | 4000 |
| NADH | | | 38-145 | 50 | 200 |

| metabolites | Buchholz 2001 *(16)* | Peng et al. 2004 *(15)* | Sauter et al. 2004 *(14)* | Jovanovic et al. 2006 *(13)* | Bennett et al. 2009 *(12)* |
|---|---|---|---|---|---|
| Glucose | | | | | |
| G6P | 220 | 680-1213 | 170 | | |
| F6P | 250 | 290-480 | 40 | | |
| FBP | 3290 | 1010-3100 | | | 15000 |
| DHAP | | | | | 370 |
| GAP | | | | | |
| BPG | | | | 60-250 | |
| 3-PG | | | | | 1500 |
| 2-PG | | | | | |
| PEP | | 70-200 | 600 | | 180 |
| Pyruvate | | | 400 | | |
| ATP | 310 | | | | 9600 |
| ADP | 400 | | | | 560 |
| $P_i$ | | | | | |
| $NAD^+$ | 3550 | | | | 2600 |
| NADH | | | | | 83 |

Table S3: Gibbs energy of reactions of glycolysis $\boldsymbol{\Delta^R g}$ at pH 7. Calculated using ePC-SAFT with parameters from Tables 2 and 3, $\boldsymbol{\Delta^R g^0}$ and $\boldsymbol{\Delta^R h^0}$ from Table 1 and metabolite concentrations from Table S2. Best-case concentrations for all reactions were used.

| | reaction conditions **A** | | reaction conditions **B** | |
|---|---|---|---|---|
| reaction | $\Delta^R g$(298.15 K) kJ mol$^{-1}$ | $\Delta^R g$(310.15 K) kJ mol$^{-1}$ | $\Delta^R g$(298.15 K) kJ mol$^{-1}$ | $\Delta^R g$(310.15 K) kJ mol$^{-1}$ |
| Hexokinase | -41.7 | -42.5 | -41.8 | -42.6 |
| G6P isomerase | -8.1 | -9.0 | -9.0 | -9.8 |
| PFK | -30.8 | -31.8 | -37.7 | -39.1 |
| Aldolase | -21.7 | -33.7 | -19.9 | -22.8 |
| TPI | -3.4 | -4.2 | -5.5 | -6.6 |
| GAPDH | **0.3** | **0.1** | -10.6 | -11.4 |
| PGK | -22.5 | -21.4 | -12.4 | -10.7 |
| PGAM | -18.2 | -19.0 | -18.2 | -19.0 |
| Enolase | -4.2 | -5.4 | -4.4 | -5.3 |
| PK | -36.9 | -38.0 | -35.5 | -36.6 |

A: considering substrates and products of the respective reaction for prediction of $\boldsymbol{Q_\gamma}$

B: considering all metabolites involved in glycolysis pathway (best-case concentrations for reacting agents + maximum concentration for the other metabolites within glycolysis, see Table S4) and 1 mmol kg$^{-1}$ $MgCl_2$ and 100 mmol kg$^{-1}$ KCl for prediction of $\boldsymbol{Q_\gamma}$

Table S4: Minimum and maximum metabolite concentrations in μM used for TFA (molarities assumed to be equal to molalities for any calculation). See Table S2 for sources.

| metabolites | minimum | maximum |
|---|---|---|
| Glucose | 5000 | 5000 |
| G6P | 38.5 | 1213 |
| F6P | 14 | 480 |
| FBP | 7 | 15000 |
| DHAP | 17 | 370 |
| GAP | 5.7 | 18.5 |
| BPG | 0.4 | 250 |
| 3-PG | 68.5 | 1500 |
| 2-PG | 10 | 29.5 |
| PEP | 17 | 600 |
| Pyruvate | 51 | 400 |
| ATP | 310 | 9600 |
| ADP | 75 | 1040 |
| $P_i$ | 500 | 7900 |
| $NAD^+$ | 650 | 4000 |
| NADH | 38 | 200 |

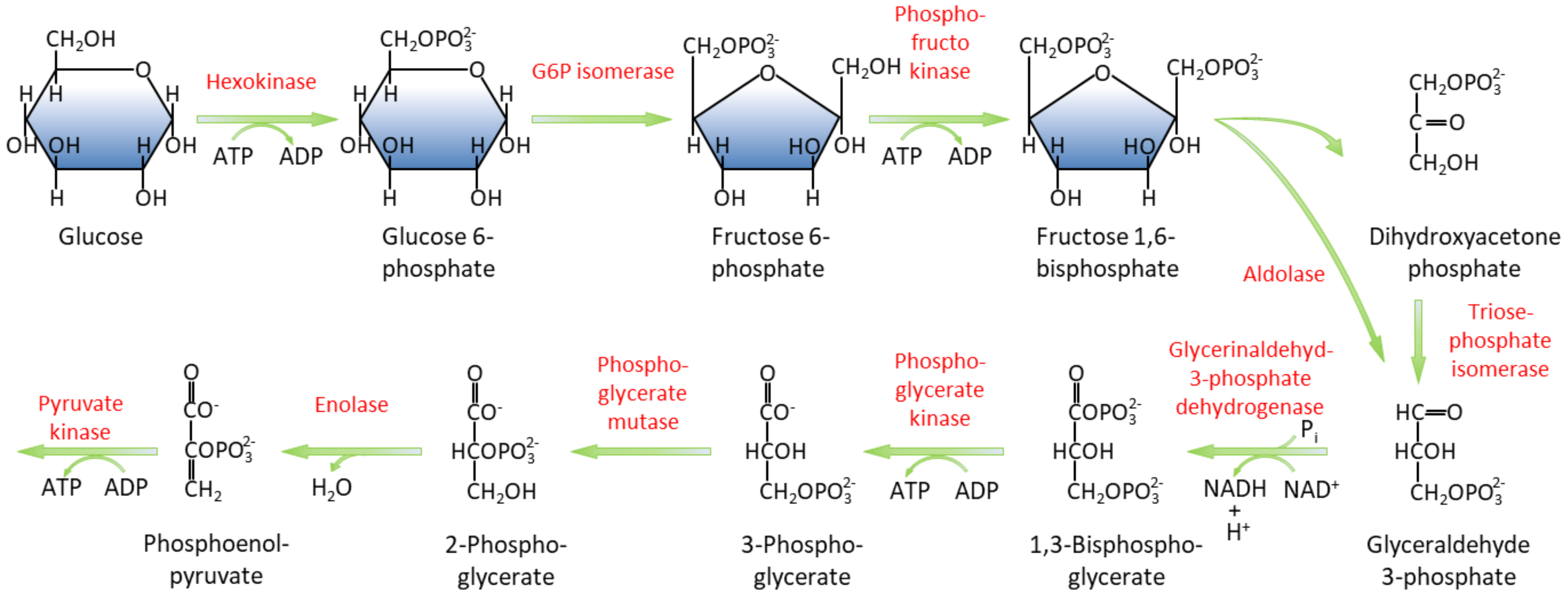


Figure S1: Schematic representation of the reaction steps of glycolysis. Enzymes in red text.

# References


1. Alberty, R. A. Thermodynamics of biochemical reactions, Wiley-Interscience: Hoboken, N.J, 2003.

2. García, A. B., Cameselle, J., García Barros, F. J., Higes Rolando, F. J., Calahorro, C. V., and Claros, J. C. V. Interaction of divalent metal ions with fructose 2,6-bisphosphate and analogs, *Journal of Inorganic Biochemistry.* **1996**, *62,* 57–65.

3. Stephanopoulos, G. N., Aristidou, A. A., and Nielsen, J. H. Metabolic engineering. Principles and methodologies, Acad. Press: San Diego, CA, 2008.

4. Pissarra, P. D. N., and Nielsen, J. Thermodynamics of Metabolic Pathways for Penicillin Production. Analysis of Thermodynamic Feasibility and Free Energy Changes During Fed-Batch Cultivation, *Biotechnol. Prog.* **1997**, *13,* 156–165.

5. Führer, L., Kubicek, C. P., and Röhr, M. Pyridine nucleotide levels and ratios in *Aspergillus niger*, *Can. J. Microbiol.* **1980**, *26,* 405–408.

6. Torres, N. V. Modeling approach to control of carbohydrate metabolism during citric acid accumulation by *Aspergillus niger*: I. Model definition and stability of the steady state, *Biotechnol. Bioeng.* **1994**, *44,* 104–111.

7. Maskow, T., and von Stockar, U. How reliable are thermodynamic feasibility statements of biochemical pathways?, *Biotechnol. Bioeng.* **2005**, *92,* 223–230.

8. Jaklitsch, W. M., Hampel, W., Röhr, M., Kubicek, C. P., and Gamerith, G. alpha-Aminoadipate pool concentration and penicillin biosynthesis in strains of *Penicillium chrysogenum*, *Can. J. Microbiol.* **1986**, *32,* 473–480.

9. Müller, R. H., Loffhagen, N., and Babel, W. Rapid extraction of (di)nucleotides from bacterial cells and determination by ion-pair reversed-phase HPLC, *J. Microbiol. Meth.* **1996**, *25,* 29–35.

10. Lehninger, A. L. Biochemistry, Worth: New York, NY, 1981.

11. Minakami, S., and Yoshikawa, H. Studies on Erythrocyte Glycolysis II. Free Energy Changes and Rate Limitings Steps in Erythrocyte Glycolysis, *J. Biochem.* **1966**, *59,* 139–144.

12. Bennett, B. D., Kimball, E. H., Gao, M., Osterhout, R., van Dien, S. J., and Rabinowitz, J. D. Absolute metabolite concentrations and implied enzyme active site occupancy in *Escherichia coli*, *Nat. Chem. Biol.* **2009**, *5,* 593–599.

13. Jovanović, S., Jovanović, N., and Jovanović, A. High glucose protects single beating adult cardiomyocytes against hypoxia, *Biochem. Bioph. Res. Co.* **2006**, *341,* 57–66.

14. Sauter, T., and Gilles, E. D. Modeling and experimental validation of the signal transduction via the *Escherichia coli* sucrose phospho transferase system, *J. Biotechnol.* **2004**, *110,* 181–199.

15. Peng, L., Arauzo-Bravo, M. J., and Shimizu, K. Metabolic flux analysis for a ppc mutant *Escherichia coli* based on $^{13}$C-labelling experiments together with enzyme activity assays and intracellular metabolite measurements, *FEMS Microbiol. Lett.* **2004**, *235,* 17–23.

16. Buchholz, A., Takors, R., and Wandrey, C. Quantification of intracellular metabolites in *Escherichia coli* K12 using liquid chromatographic-electrospray ionization tandem mass spectrometric techniques, *Anal. Biochem.* **2001**, *295,* 129–137.
